# Comment on van Casteren *et al*. (2018): softer metallic spheres do abrade harder enamel

**DOI:** 10.1098/rsos.181376

**Published:** 2018-11-28

**Authors:** Jing Xia, Zhongrong Zhou, Linmao Qian, Peter S. Ungar

**Affiliations:** 1 Tribology Research Institute, Key Laboratory of Advanced Technologies of Materials, Ministry of Education, Southwest Jiaotong University, Chengdu 610031, People's Republic of China; 2 Department of Anthropology, University of Arkansas, Fayetteville, AR 72701, USA

## 1. Introduction

Xia *et al*. recently [1,2] presented experimental evidence that dental enamel is abraded by particles softer than this tissue. These results have important implications for paleontologists, biotribologists and dental clinicians alike. It was argued that because enamel crystallites are glued together by proteins, tissue removal requires only that contact pressure be sufficient to break the protein bonds holding enamel nanofibres together, so that materials softer than enamel (i.e. aluminium and brass) can and do transmit such contact pressures. This is consistent with previous reports that microfracture of enamel can occur without plastic deformation [3], and that friction readily leads to wear debris with microcrack propagation [4].

Van Casteren *et al*. [5], however, recently suggested in this journal that Xia *et al*.'s model was overly simplistic, and that the metallic spheres used in their study were actually harder than enamel, and therefore are consistent with a previous mechanical model [6] suggesting that softer materials do not wear tooth crowns. Van Casteren *et al*. argued that the aluminium balls used by Xia *et al*. were surfaced by a thin rough oxide layer harder than enamel, and that the brass ball surfaces actually have hardness values comparable to or higher than enamel due to work hardening during manufacture. We here show that van Casteren *et al*.'s calculations of sphere hardness are erroneous and that their experimental results are actually in line with Xia *et al*.'s original arguments. We also present results of new experiments that offer additional empirical evidence to confirm Xia *et al*.'s original assertion that aluminium and brass metallic balls are softer than dental enamel, yet still abrade the harder tissue.

## 2. Errors in hardness estimates by van Casteren *et al.*

Van Casteren *et al*. indicate that in their study of aluminium spheres, ‘average contact pressures typically rose above 5 GPa… to levels greater than 10 GPa’ [5]. These values are erroneous because the authors used the wrong formula to calculate them. The authors indicated, ‘the radius of contact *a* in the plane of the oxide surface can be approximated as *a* = (*Rd*)^0.5^, where *R* is the radius of the indenter tip’ [5]. They continued, ‘the area of contact is *πa*^2^, thus giving an expression for the calculated average pressure of contact as *F*/*πdR*, where *F* is the force’ [5].

**Figure 1.**
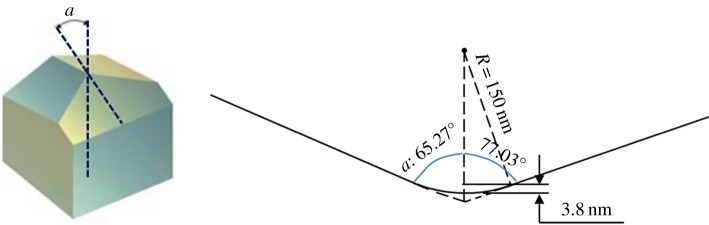
Effective contact depth within the spherical part of the Berkovich tip with radius *R* = 150 nm.

The formula *a* = (*Rd*)^0.5^ is correct for radius of contact only when the indentation process is fully elastic. However, as is evident from figure 1*b* in van Casteren *et al*., three of four indentations with maximum depths of 45–50 nm in their experiments have residual indentation depths of 30–35 nm. Hernot *et al*. [7] demonstrated that *a*^2^ = 2*c*^2^*dR*, where *c*^2^ = 0.5 for elastic indentation. However, when the stress under the indenter is higher than the yield stress of the indented material, *c*^2^ increases with indentation depth during the elastic–plastic indentation phase. For higher indention depths, *c*^2^ is again constant during the fully plastic phase. Therefore, the estimated average contact pressure reported by the authors is larger than the actual contact pressure.

This error is compounded by incorrect assumptions regarding calculations given the irregular shape of the Berkovich diamond indentation tip. The pointed end of a Berkovich tip cannot be considered spherical given manufacturing tolerances and wear of the tip. The shape of an indenter tip is typically calibrated on fused quartz samples, but this process can only produce an area-depth curve—it cannot be used to calculate curvature radius. This makes it impossible to confirm that the radius tip is (as reported) 150 nm.

The Berkovich tip has an included angle of 142.3° and a half angle *a* = 65.27°, measured from the axis to one of the pyramid flats. Therefore, the angle of that axis relative to the pyramid ridge is 77.03° (figure 1). Even if the curvature radius of the tip was reported correctly by van Casteren *et al*., as *R* = 150 nm, the valid contact depth *d* for calculating average contact pressure, which is calculated as *d* = *R* × [1 − sin(77.03°)] is approximately 3.8 nm. Van Casteren *et al*.'s estimate of contact pressure is invalid also because they use radius *R* = 150 nm at a depth larger than 3.8 nm (see van Casteren *et al*., figure 1*c*). For a pristine, perfectly tooled Berkovich tip, the contact area at a depth larger than 3.8 nm can be calculated as *A* = [*d* × tan(77.03°)]^2^ × 1.5 × cos(30°). Hence, with a contact depth of 50 nm, the contact area estimate of van Casteren *et al*. is about one-third the actual contact area, so the average contact pressure reported by the authors is 3x higher than the actual value and the resulting estimates of hardness are grossly inaccurate.

We note also that Berkovich tips are blunted with use. Therefore, the formula

$$A=24.5h_{c}^{2}+C_{1}h_{c}+C_{2}h_{c}^{1/2}+C_{3}h_{c}^{1/4}+C_{4}h_{c}^{1/8}+C_{5}h_{c}^{1/16}$$

should have been used to calculate the contact pressure. The lead term $24.5h_{c}^{2}$ describes a perfect Berkovich indenter, whereas the other terms describe deviations from Berkovich geometry due to blunting at the tip [8]. Values for *C*_1_ through *C*_5_ are calculated typically by the nanoindentor system during the process of tip calibration (see below).

## 3. Experimental/empirical verification

Results from Xia *et al*. [2] were verified with further experimental study using the methods described in that original paper. Brass (1.8 mm diameter) and aluminium (3.0 mm diameter) spheres were obtained from the same manufacturer as those used in the original studies by both Xia *et al*. and van Casteren *et al*. Indentation hardness of these spheres was determined by a nanoindentation tester (T750, Hysitron Inc., Eden Prairie, MN, USA) using a Berkovich diamond tip. Before the indentation tests, the shape of the tip was calibrated using a fused quartz sample, the values of *C*_1_ through *C*_5_ were given by the system as: *C*_1_ = 1.9217 × 10^4^, *C*_2_ = −1.0432 × 10^6^, *C*_3_ = −2.1670 × 10^7^, *C*_4_ = −2.1670 × 10^7^, *C*_5_ = 1.3398 × 10^7^. To confirm the accuracy of hardness measurements, the hardness of enamel was tested. Thirty indentations were made on enamel with a maximum indentation force of 250 µN. The hardness of our enamel sample was measured as 4.82 ± 0.15 GPa, which is in accordance with values reported previously in the literature [9]. Both aluminium and brass spheres were indented orthogonal to their outer surfaces with a maximum indentation force of 250 µN. Ten sets of indentations were made on each of three aluminium balls and three brass balls. In addition, indentations were made at a maximum indentation force of 50 µN, 250 µN, 500 µN, 1 mN and 8 mN on the aluminium balls and 100 µN, 250 µN, 2 mN, 5 mN and 8 mN on the brass balls to calculate hardness of both the outer and inner layers of each.

**Figure 2.**
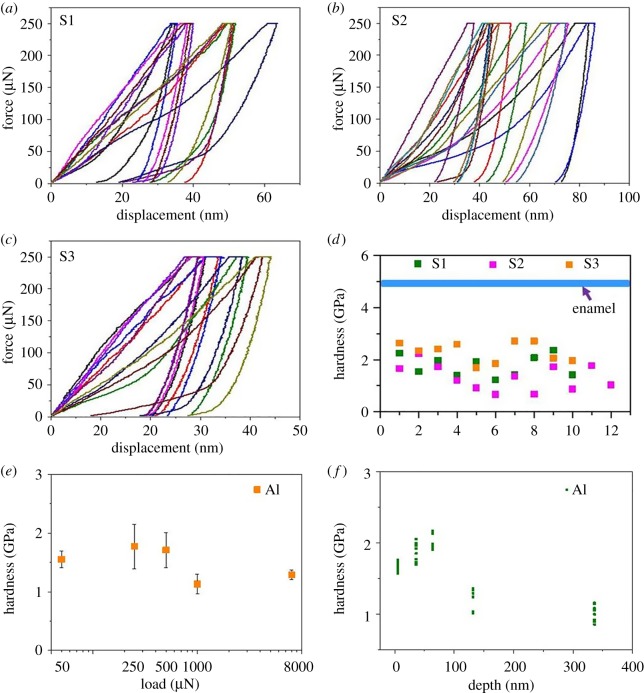
Detection of force–displacement curves and hardness on the outer layer surface of three aluminium balls at a maximum indentation force of 250 µN. (*a*) Force–displacement curves of sample 1 (S1); (*b*) force–displacement curves of sample 2 (S2); (*c*) force–displacement curves of sample 3 (S3); (*d*) corresponding hardness of the three samples. The hardness of all the indentations measured in the aluminium balls was lower than enamel (blue line). Hardness values for aluminium balls under different normal loads and depths are illustrated in (*e*) and (*f*), respectively. See Xia *et al*. [2] for methods.

Figure 2*a*–*d* shows nanoindentation force–displacement curves and corresponding hardness values for the three aluminium balls under a maximum indentation force of 250 µN. The maximum indentation depth for each indentation curve ranged from 27 to 80 nm, and indentation hardness was determined by the instrument with a range from 0.67 to 2.7 GPa. These values are all much lower than those reported for enamel. Hardness values of the aluminium balls at given normal loads and depths are illustrated in figure 2*e*,*f*. Although the hardness of the outer layer was indeed higher than that for the inner layer, both were much lower than those of enamel.

**Figure 3.**
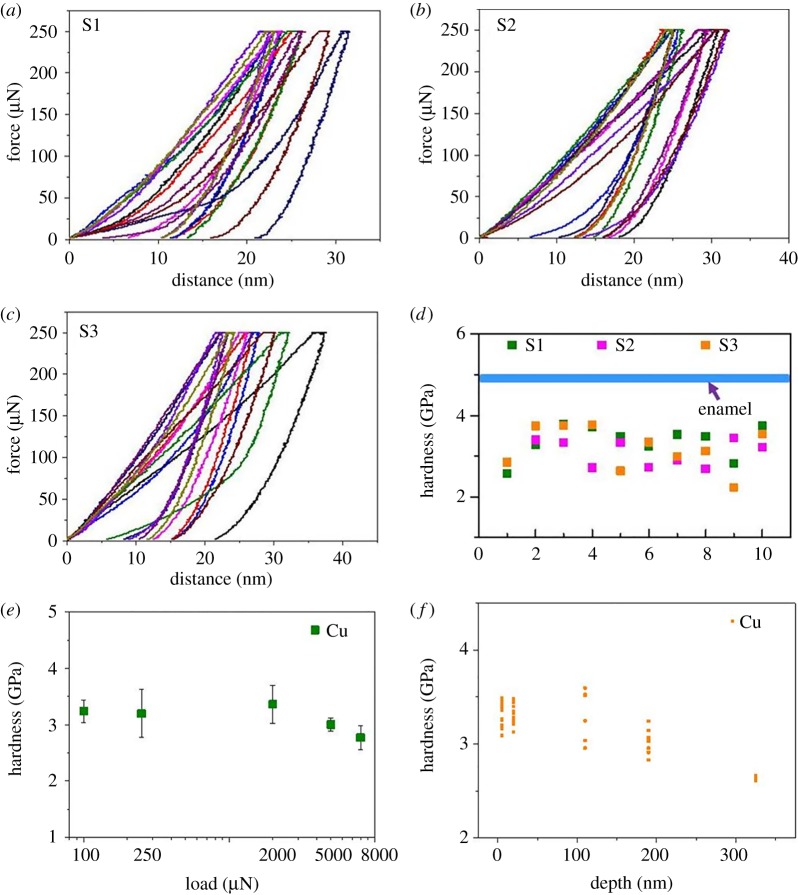
Detection of force–displacement curves and hardness on the outer layer surface of three brass balls at a maximum indentation force of 250 µN. (*a*) Force–displacement curves of sample 1 (S1); (*b*) force–displacement curves of sample 2 (S2); (*c*) force–displacement curves of sample 3 (S3); (*d*) corresponding hardness of the three samples. The hardness of all the indentations measured in the brass balls was lower than enamel (blue line). Hardness values for brass balls under different normal loads and depths are illustrated in (*e*) and (*f*), respectively. See Xia *et al*. [2] for methods.

**Figure 4.**
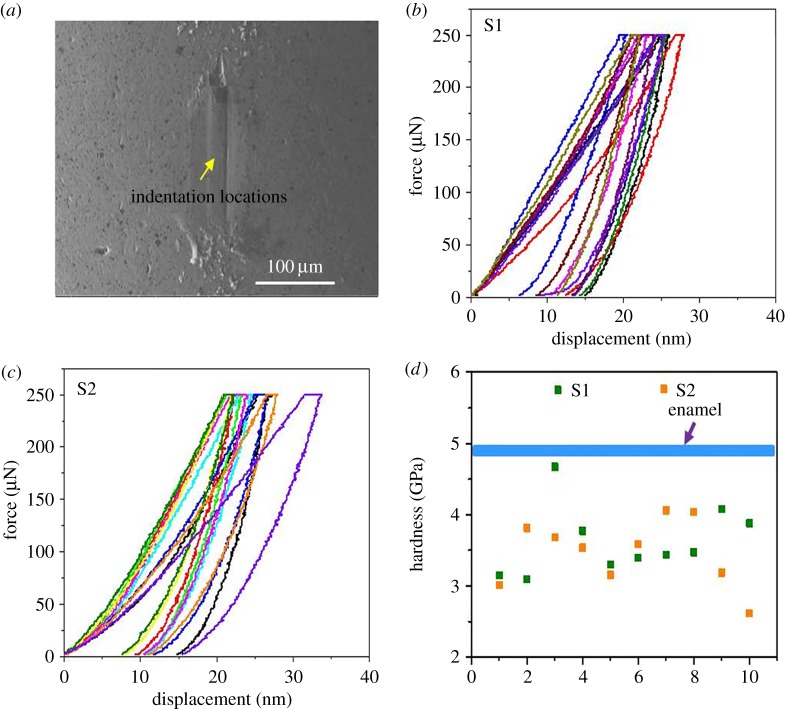
Detection of force–displacement curves and corresponding hardness of two brass balls after 60 cycles scratching against enamel at a maximum indentation force of 250 µN. (*a*) Indentations were located in the scratch area on the brass balls; (*b*) force–displacement curves of sample 1; (*c*) force–displacement curves of sample 2; (*d*) corresponding hardness of the two samples. The hardness of each of the brass balls was again lower than enamel (blue line). See Xia *et al*. [2] for methods.

Figure 3*a*–*d* shows nanoindentation force–displacement curves and corresponding hardness values for the three brass balls under a maximum indentation force of 250 µN. The hardness of these brass balls ranged from 2.23 to 3.79 GPa. Hardness values of the brass balls at given normal loads and depths are illustrated in figure 3*e*,*f*. Although the hardness of the outer layer was again higher than that for the inner layer, both were also much lower than those of enamel. Even after 60 scratch cycles against enamel (figure 4), the hardness of the brass balls ranged only from 2.61 to 4.08 GPa—suggesting that work hardening does not make these balls harder than enamel.

**Figure 5.**
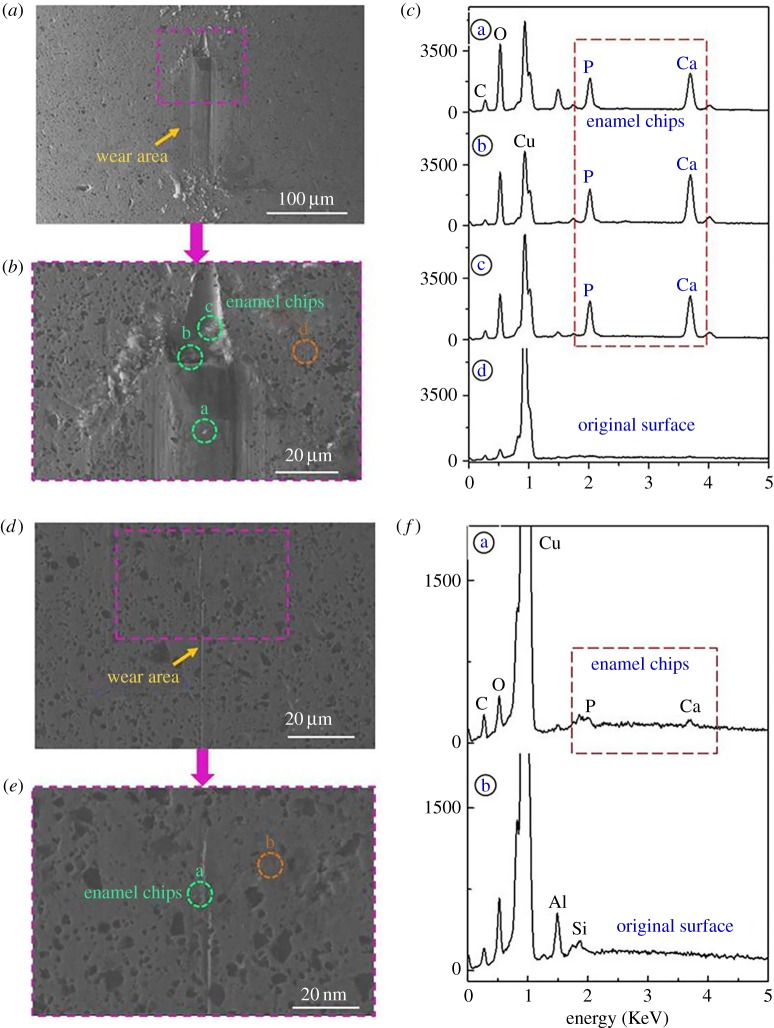
Enamel chips embedded in the surface of a brass ball after 60 cycles (*a*–*c*) and one cycle (*d*–*f*) of scratching under contact pressure of 1.61 GPa. SEM images indicate locations of enamel debris on the brass ball confirmed by the Ca and P peaks in the EDX spectrum. See Xia *et al*. [2] for methods.

To confirm not only that metallic balls are softer than enamel but that they can and do wear the tissue, we conducted further scratch tests on enamel using these same spheres following the method described in Xia *et al*. [2]. Experiments revealed chips clearly embedded in the spheres, demonstrating beyond doubt that softer metallic spheres can and do abrade harder enamel (figure 5). Indeed, enamel chips are detected after even a single scratch cycle, clearly indicating that the effect is not a result of work hardening or fatigue wear. This is consistent with the notion that enamel wear is not predicated on abrasive hardness but, rather, on contact pressure sufficient to break the protein bonds that hold nanoparticles to the surface. It is possible that plastic deformation of the metallic spheres under contact load, along with metal fragments at the sliding interface, contributed to the wear observed.

**Figure 6.**
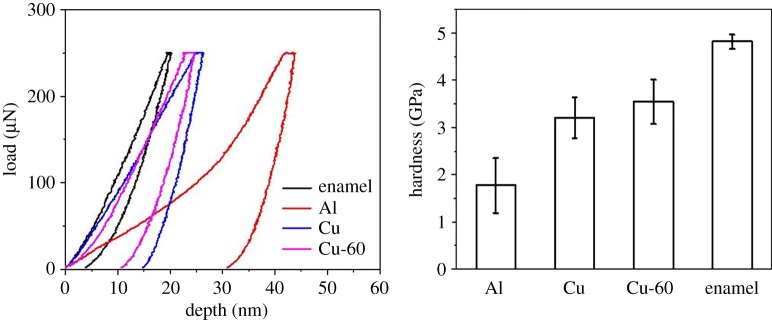
Comparisons of the indentation curves and hardnesses of enamel, aluminium ball, brass ball, and brass ball after 60 cycles drag.

Finally, to compare directly the hardness of the aluminium balls, brass balls, and tooth enamel, these materials were all exposed to the same indentation loading conditions (figure 6). Results again clearly show that even under a very low indentation load (250 µN), the indentation hardness values of aluminium spheres, brass spheres, and brass spheres after 60 scratch cycles are all much smaller than that of enamel.

## Supplementary Material

Supplemental Data file-Raw data

## Data accessibility

All data gathered for this study are presented in the text, figures and electronic supplementary materials.
