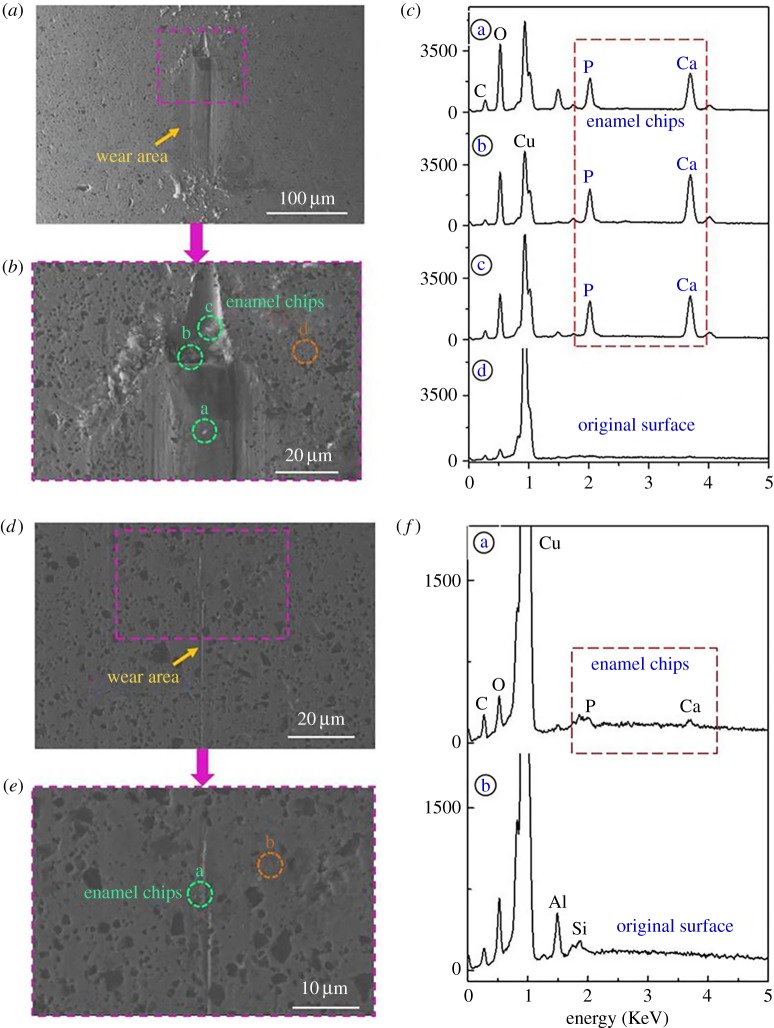# Correction to: ‘Comment on van Casteren *et al.* (2018): softer metallic spheres do abrade harder enamel’

**DOI:** 10.1098/rsos.190229

**Published:** 2019-02-20

**Authors:** Jing Xia, Zhongrong Zhou, Linmao Qian, Peter S. Ungar

*R. Soc. open sci.* **5**, 181376. (Published 1 November 2018). (doi:10.1098/rsos.181376)

The authors wish to correct two misprints in the original article:

1. The scale bar for Figure 5*e* represents 10 µm. The corrected figure is shown below.
2. In line 7 of the experimental/empirical verification section, the value reported for C_3_ should be 9.319×10^6^.

**Figure:**